# Time scales of autonomic information flow in near-term fetal sheep

**DOI:** 10.3389/fphys.2012.00378

**Published:** 2012-09-21

**Authors:** M. G. Frasch, B. Frank, M. Last, T. Müller

**Affiliations:** ^1^CHU Sainte-Justine Research Centre, Department of Obstetrics and Gynecology, Faculty of Medicine, Université de MontréalQC, Canada; ^2^Centre de recherche en reproduction animale (CRRA), Faculty of Veterinary Medicine, Université de MontréalQC, Canada; ^3^Biomagnetic Centre, Department of Neurology, Friedrich Schiller UniversityJena, Germany; ^4^School of Medicine, University of California, DavisCA, USA; ^5^Institute of Laboratory Animal Science, Friedrich Schiller UniversityJena, Germany

**Keywords:** fetal heart rate, HRV, autonomic nervous system, permutation entropy, complexity, sleep, atropine, propranolol

## Abstract

Autonomic information flow (AIF) characterizes fetal heart rate (FHR) variability (fHRV) in the time scale dependent complexity domain and discriminates sleep states [high voltage/low frequency (HV/LF) and low voltage/high frequency (LV/HF) electrocortical activity (ECoG)]. However, the physiologic relationship of AIF time scales to the underlying sympathetic and vagal rhythms is not known. Understanding this relationship will enhance the benefits derived from using fHRV to monitor fetal health non-invasively. We analyzed AIF measured as Kullback–Leibler entropy (KLE) in fetal sheep in late gestation as function of vagal and sympathetic modulation of fHRV, using atropine and propranolol, respectively (*n* = 6), and also analyzed changes in fHRV during sleep states (*n* = 12). Atropine blockade resulted in complexity decrease at 2.5 Hz compared to baseline HV/LF and LV/HF states and at 1.6 Hz compared to LV/HF. Propranolol blockade resulted in complexity increase in the 0.8–1 Hz range compared to LV/HF and in no changes when compared to HV/LF. During LV/HF state activity, fHRV complexity was lower at 2.5 Hz and higher at 0.15–0.19 Hz than during HV/LF. Our findings show that in mature fetuses near term vagal activity contributes to fHRV complexity on a wider range of time scales than sympathetic activity. Related to sleep, during LV/HF we found lower complexity at short-term time scale where complexity is also decreased due to vagal blockade. We conclude that vagal and sympathetic modulations of fHRV show sleep state-dependent and time scale-dependent complexity patterns captured by AIF analysis of fHRV. Specifically, we observed a vagally mediated and sleep state-dependent change in these patterns at a time scale around 2.5 Hz (0.2 s). A paradigm of state-dependent non-linear sympathovagal modulation of fHRV is discussed.

## Introduction

Autonomic vagal and sympathetic activities in sheep and human fetuses vary with behavioral states and with the health of the fetus. This has been shown non-invasively in human fetuses and in ovine models of human fetal development by analysis of the fetal heart rate (FHR) variability (fHRV) (Karin et al., 1993; Groome et al., 1994; Metsala et al., 1995; Kimura et al., 1996; Van Leeuwen et al., 2003; Frank et al., 2006a,b; Schneider et al., 2008, 2009; Frasch et al., 2009a,b; Hoyer et al., 2009; Lange et al., 2009; van Laar et al., 2011). In the sheep fetus in late gestation, electrocortical activity (ECoG) is characterized by the alternating high voltage/low frequency (HV/LF) and low voltage/high frequency (LV/HF) states. The HV/LF states are similar to the human 1F state, while the LV/HF states are similar to 2F in the human fetus (Frank et al., 2006a,b; Keen et al., 2011a,b).

FHRV holds promise as a non-invasive, continuous, sensitive, and specific measure that may identify fetuses at risk of adverse outcomes and requiring intervention. Methods assessing linear properties of fHRV to estimate the sympathetic and vagal modulation of fHRV enhanced our knowledge of fetal physiology and pathophysiology (Karin et al., 1993; Groome et al., 1994; Metsala et al., 1995; Kimura et al., 1996; Van Leeuwen et al., 2003; van Laar et al., 2011). However, due to the fundamentally non-linear structure of HRV additional methods are required in order to capture the non-linear fHRV properties, thus overcoming the methodical limitation of the linear HRV analysis (Groome et al., 1999; Frasch et al., 2009a,b; Hoyer et al., 2011).

The origin of the non-linearity of sympathovagal interactions lies in their intrinsic complexity which emerges from interaction of neuronal brainstem networks as weakly coupled non-linear oscillators that are influenced by various afferent signals (Szeto et al., 1992; Fleisher et al., 1997; Lambertz et al., 2000; Vandenhouten et al., 2000). Our knowledge on the impact of the sympathovagal activities on fHRV complexity and non-linearity is still very limited (Hoyer et al., 2005; Frasch et al., 2009a,b; Cysarz et al., 2011; Doret et al., 2011; Hoyer et al., 2011). Specifically, while the influence of the vagal and sympathetic modulations on fHRV properties is non-linear and dependent on the time scale of observation (Frasch et al., 2009a,b; Hoyer et al., 2011), the time scale-dependent changes in fHRV complexity during physiological electrocortical state activity or under conditions of pharmacological vagal or sympathetic blockade are unknown.

Consequently, our objective was to assess the contribution of vagal and sympathetic activities to *all* time scales of fHRV complexity during HV/LF and LV/HF states in near-term fetus. To this end, we studied fHRV complexity parameters derived from Kullback–Leibler entropy (KLE). KLE has been introduced and validated in human and sheep fetuses (Frank et al., 2006a,b). Briefly, decreasing KLE values correspond to increasing fHRV complexity until they reach zero for noise. KLE's main features are (1) robustness with respect to some noise possibly corrupting the data as is often the case in real life fHRV, and (2) easy computation. Thus, KLE appears to be particularly well suited for fHRV computation offline as well as for potential future development of online fHRV analysis tools (Frasch, 2011). Moreover, in agreement with the non-linear nature of sympathovagal interactions and our objective, computation of KLE over *all* time scales allowed us to make no *a priori* assumptions as to the physiologically relevant “temporal cross sections” through KLE function describing fHRV complexity. We thus captured all potentially relevant fHRV complexity changes dependant on the pharmacological blockades or behavioral ECoG states.

## Methods

### Surgical procedure

Experimental procedures were approved by the animal welfare commission of Thuringia. Thirteen Long-Wool Merino × German Blackheaded Mutton cross-bred ewes of known gestational age were acclimated to the animal facilities for at least 5 days before surgery. After food withdrawal for 24 h, surgery was performed under halothane general anesthesia. Following 1 g of ketamine (Ketamin 10, Atarost, Germany) i.m., anaesthesia was induced by 4% halothane (Halothane Liquid 250 ml, Rhodia Organique Fine Ltd., UK) using a face mask. Ewes were intubated and anaesthesia was maintained with 1.0–1.5% halothane in 100% oxygen. Ewes were instrumented with catheters inserted into the common carotid artery for blood sampling and into the external jugular vein for post-operative administration of drugs.

Following hysterotomy, fetuses were instrumented with polyvinyl catheters (Rüschelit, Rüsch, Germany) inserted into the left common carotid artery for arterial blood pressure (ABP) recordings and blood sampling and into the left external jugular vein for drug administration. An additional catheter was placed in the amniotic cavity to record the amniotic pressure in order to permit correction of fetal mean ABP for hydrostatic pressure. Wire electrodes (LIFYY, Metrofunk Kabel-Union, Germany) were implanted into the left suprascapular muscles, muscles of the right shoulder and in the cartilage of the sternum for electrocardiogram (ECG) recording, into the uterine wall to record myometrial activity and into the skull to record electrocorticogram (ECoG) as bihemispherial leads from frontal and parietal regions and fixed with dental cement on the skull bone.

All ewes and fetuses received 0.5 g ampicillin (Ampicillin, Ratiopharm, Germany) intravenously and into the amniotic sac twice a day during the first 3 post-operative days. Metamizol (Arthripur, Atarost, Germany) was administered intravenously to the ewe (30–50 mg·kg^−1^) as an analgesic for at least 3 days. All catheters were maintained patent via a continuous infusion of heparin at 15 IU·ml^−1^ in 0.9% saline delivered at 0.5 ml·h^−1^.

### Experimental protocol

After at least 3 days of post-operative recovery, the experimental protocol started at 09:00 a.m. In seven sheep, at 127 ± 3 days gestational age (dGA, term 150 days) ECG, ECoG, ABP, and uterine EMG were recorded continuously for the duration of the whole experiment. Arterial blood samples were taken daily at 09:00. The samples were analyzed for fetal blood gases, hemoglobin concentration, and oxygen saturation using a blood gas analyzer (ABL600, Radiometer, Denmark; measurements corrected to 39°C). We reported these data to be within physiological range (Frasch et al., 2009a,b).

Five minute ECG epochs were selected in HV/LF and LV/HF ECoG, since at this gestational age sleep state cycling is developed (Frank et al., 2006a,b). Sleep states were determined from ECoG visually and confirmed quantitatively by means of spectral edge frequency analysis of the bifrontal ECoG. This group of fetuses received 2.5 mg atropine-sulfate (Atropinsulfat, B. Braun, Melsungen, Germany) intravenously as a 5 ml bolus to induce vagal blockade and, 24 h later, 2 mg propranolol (Obsidan, Alpharma-Isis, Langenfeld, Germany) as a 2 ml bolus over 60 s to induce a beta-receptor mediated sympathetic blockade according to Yu et al. (Yu and Lumbers, 2000). Starting five minutes after the injections, ECG was analyzed over five minutes.

### Data acquisition

ABP and amniotic pressure were recorded continuously using calibrated pressure transducers (B. Braun, Germany). Myometrial activity was monitored to recognize pressure artifacts during contractures. All biophysical parameters were amplified (Amplifier Model 5900 and 6600, Gould, USA) and digitized using a 16-channel A/D board (DI-400-PGH, DATAQ Instruments, USA) at a sample rate of 1000 s^−1^ for ECG and 100 s^−1^ for blood pressures and uterine EMG and continuously stored on a hard disc of a PC.

### Analysis of physiological variables

The software package Matlab 6.1, R13, was used to calculate all fHRV measures (The MathWorks, Natick, MA, USA). First, for calculation of FHR and fHRV the individual R peaks were sequentially detected and triggered with a precision of ± 0.49 milliseconds. The fHRV was further processed as described earlier (Frasch et al., 2007a,b, 2009a,b). Briefly, the artifacts were visually controlled for and removed manually. The resulting instantaneous R–R interval sequence was linearly interpolated at a 1000 Hz equidistant sample rate and re-sampled at 10 Hz for further signal analysis.

We reported the behavior of FHR as well as the linear fHRV measures in time and frequency domains (Frasch et al., 2009a,b). Here we aimed to characterize the behavior of the non-linear time scale properties of fHRV under physiological perturbations.

### Permutation entropy and Kullback–Leibler entropy

Permutation entropy is a complexity measure for time series operating on an ordinal level, i.e., only the ranks of the data in the time series are analyzed, not the distances (metric) of the data. Permutation entropy measures the entropy of sequences of ordinal patterns derived from *m*-dimensional delay embedding vectors. In the following we briefly summarize the definition of the permutation entropy. A more detailed introduction can be found in the references (Bandt and Pompe, 2002; Cao et al., 2004).

The scalar time series {*x*(*t*)}^*T*^_*t* = 1_ is embedded into an *m*-dimensional space *X*_*t*_ = [*x*(*t*), *x*(*t* + *L*),…, *x*(*t* + (*m* − 1)*L*], where *m* is called the embedding dimension and *L* the embedding delay time. For *m* = 2, there are two possible ordinal patterns of *X*_*t*_, namely π_1_ = *x*(*t*) < *x*(*t* + *L*) and π_2_ = *x*(*t* + *L*) < *x*(*t*). (For this moment we suppose that there are no equal values in *X*_*t*_, i.e., no tied ranks.) For *m* = 3, *X*_*t*_ can attain one of six different order patterns,

$$\begin{array}{c} \pi_{1}=x(t)<x(t+L)<x(t+2L),\\ {\text{ π}}_{\text{2}}=x(t+L)<x(t)<x(t+2L),\ldots ,\\ \pi_{6}=x(t+2L)<x(t+L)<x(t). \end{array}$$

In general, there are just *m*! possible order patterns, which is the number of permutations of the *m* coordinates in *X*_*t*_. Now, let *p*(π) denote the relative frequency of order pattern π,

(1) $$p(\pi )=\frac{\#\left\{t|1\leq t\leq T-(m-1)L,\text{ where }X_{t}\text{ has type }\pi \right\}}{T-(m-1)L}$$

Then, for fixed embedding dimensions *m* ≥ 2, and fixed delay *L*, permutation entropy is defined as:

(2) $$H(m;L)=-\sum_{\pi }p(\pi )\log_{2}p(\pi ),$$

where the sum runs over all *m*! patterns π.

Equal values in the time series, which can occur because of the limited accuracy of measurement, will be treated as follows. In case, *X*_*t*_ contains two equal values *x*_*a*_ (*t* + *aL*) = *x*_*b*_ (*t* + *bL*), *a*,*b* = 0, 1,…, (*m* − 1), the relative frequency of the permutations which correspond to the cases *x*_*a*_ < *x*_*b*_ and *x*_*a*_ > *x*_*b*_ is increased by 1/2. For *n* equal values the respective *n*! permutations are increased by 1/*n*!. Practically this can be done by adding a random number to the data, which is smaller than the accuracy of measurements.

For convenience we normalize *H*(*m*, *L*) by its maximum value log_2_ *m*!

(3) $$0\leq H(m,L)/\log_{2}(m!)\leq 1.$$

Now we introduce the (normalized to 1) [*KLE*, Kullback, 1968]

(4) $$KLE=1-H(m,L)/\log_{2}(m!)$$

which is an information measure for the distance between the probability distribution of the ordinal patterns (permutations) and the uniform distribution. With increasing complexity of the time series, *KLE* decreases until it reaches zero for noise (independent and identically distributed (i.i.d.) process) that corresponds to a uniform distribution of all patterns. Note that due to our handling of tied ranks, a constant series would also provide *KLE* = 0.

We have to choose appropriate values for *m* and *L*. The value of *m* should be at least three; the maximum is limited by the length of time series. For an accurate estimation of *KLE*, the length of the time series must be considerably larger than the factorial of the embedding dimension. This allows for short series around 256 heartbeats only embedding dimensions *m* = 3 and *m* = 4. We tried both values and could not find significant differences in the discriminatory impact of the respective entropy measures (Frank et al., 2006a,b). Thus, based on the shortest length of time series studied, computation rate and memory requirements we chose the embedding dimension *m* = 3 in this paper. It means, in order to predict future heart beats in fHRV, three preceding heart beats from the past are taken as known information which corresponds to ED = 3. The delay time *L* is varying between 0 and 5 s. KLE of oscillators has its peaks at half the period. Thus, frequency values correspond to the time scales L as:

(5) f = 1/(2L).

Finally, to get an equidistant time scale, all data were re-sampled with a sampling frequency of 10 Hz.

### Statistical analysis

We used a method to discriminate between ECoG states based on the AIF time scale-dependent function of KLE. To avoid over-fitting due to the limited number of subjects available we used a parsimonious model with acceptable discriminatory power. The time scale-dependent variability of KLE between the animals was greater than the variability within one animal across ECoG states. This prevented the formation of global rules, such as “if KLE is above X at time scale Y, then the fetus is in LV/HF state.” Instead, we looked for rules that could be used to discriminate ECoG state of the animal when compared to other records of the same animal.

Thus, all physiological parameters were tested for differences between baseline and after drug administration or groups using the Wilcoxon or Mann–Whitney tests, respectively. Baseline LV/HF and HV/LF states were compared to the respective atropine and propranolol treatment fHRV data-set. Hence, Bonferroni–Holm correction for multiple comparisons was used. Data-sets that were 1.8 min long were used to compare the effects of atropine and propranolol blockades with the effects of HV/LF and LV/HF sleep states on fHRV complexity. This data length was chosen to be consistent in comparison of all four data-sets and, consequently, the Mann–Whitney test was used. For analyses of the 12 paired HV/LF and LV/HF data-sets, the Wilcoxon test was applied and data length could be increased to 3.5 min (as was the case in the shortest recording). All results are given as mean ± SEM. *P*-values < 0.05 were considered significant.

## Results

As reported, we found the fetal physiological parameters to be within the norm for the gestational age throughout the study (Frasch et al., 2009a,b). Mean FHR at baseline during LV/HF ECoG state was 165 ± 5 bpm which was lower than FHR during HV/LF ECoG state at 188 ± 8 bpm. Atropine administration resulted in FHR increase to 237 ± 17 bpm. Propranolol administration lead the FHR to decrease to 151 ± 4 bpm. In all cases the results were statistically significant with the exception of propranolol's effect on FHR compared to the baseline LV/HF ECoG state, which was of borderline significance (*p* = 0.052).

### Vagal and sympathetic blockades

**Figure 1:**
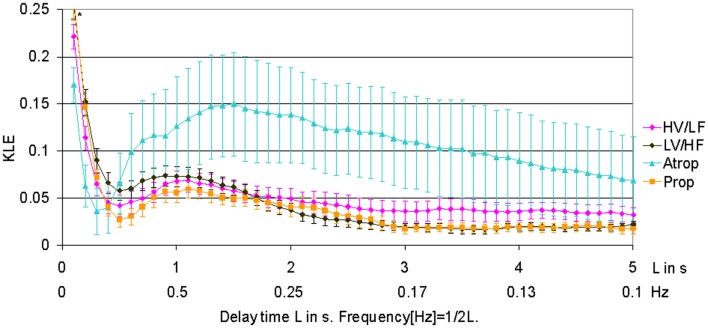
**KLE for varying delay time L (in seconds).** Increasing complexity corresponds to decreasing KLE values. The whiskers indicate the standard error of the mean. Atropine administration results in profound reduction of complexity on most of the time scales, while propranolol causes a subtle increase in complexity on time scales associated with both vagal and sympathetic modulations of fHRV. The HV/LF curve (lower complexity) lies above the LV/HF curve (higher complexity) within a long-term time scale range. *N* = 6. ^*^p < 0.05.

**Figure 2:**
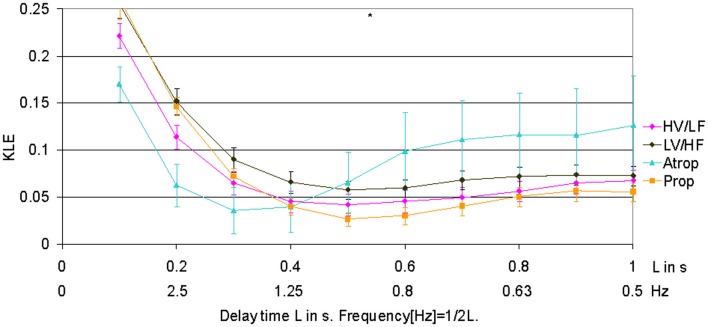
**Focus on the short-term time scale KLE segment.** This figure demonstrates changes induced by pharmacologic blockades on short-term time scale fHRV complexity measured by KLE. *N* = 6. ^*^p < 0.05.

When compared to HV/LF and LV/HF sleep states, atropine blockade resulted in an increase of KLE on the time scale of 0.2 s corresponding to a decrease in fHRV complexity in the 2.5 Hz range (Figures 1 and 2). Compared to the LV/HF state, the complexity also decreased at the 0.3 s time scale (1.6 Hz). Propranolol blockade resulted in complexity increase at 0.5–0.6 s time scales (0.8–1 Hz) compared to the LV/HF state and no changes compared to the HV/LF state (Figure 2). Figure 1 demonstrates that short (beat-to-beat) and long-term (an integral over 10 heart beats) time scale auto-autonomic information flow (aAIF) approaches to estimate fHRV complexity are relatively crude compared to the millisecond precise dissection of fHRV complexity fluctuations on each time scale that we attempted here with the KLE complexity function (Frasch et al., 2009a,b).

### HV/LF and LV/HF electrocortical state activity

**Figure 3:**
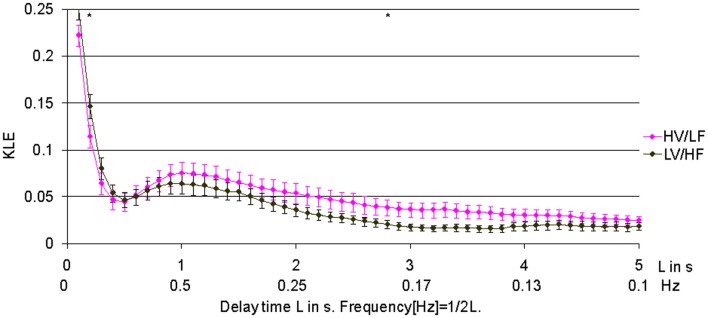
**Relationship of HV/LF and LV/HF electrocortical state activities to the time scales of fHRV complexity.** KLE complexity of fetal heart rate variability (fHRV) measured during high voltage/low frequency, HV/LF and low voltage/high frequency, LV/HF, electrocortical activites. *N* = 12, mean ± SEM. ^*^L = 3s; *p* < 0.05; *L* = 0.2s; *p* = 0.05.

**Figure 4:**
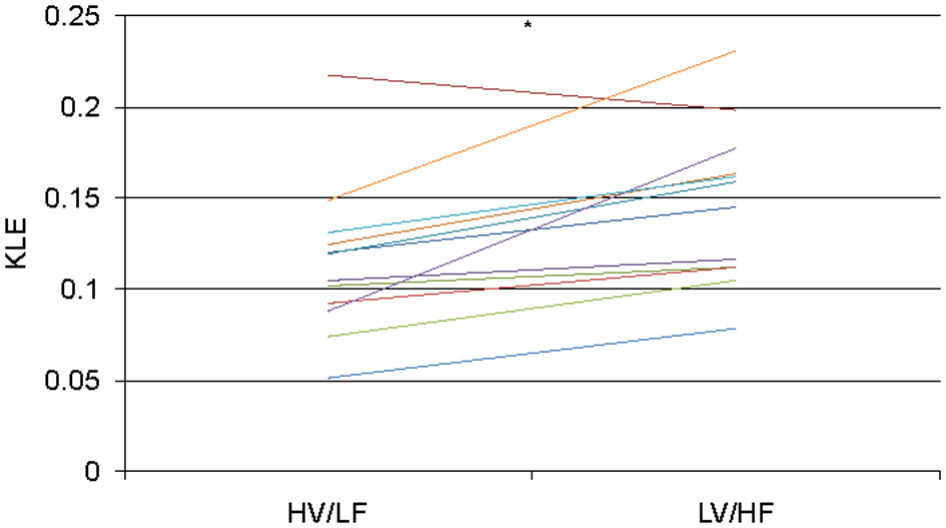
**KLE at the time scale of 2.5 Hz (0.2 s) for each animal during HV/LF versus LV/HF state.** Each line color represents a different animal. *N* = 12, ^*^p = 0.005.

In 1.8 min long data-sets used to compare effects of atropine/propranolol blockades on fHRV complexity vs. HV/LF and LV/HF sleep states no differences between the sleep states were found. We increased the data-set length to 3.5 min based on the shortest fHRV recording for the analyses of all 12 paired HV/LF and LV/HF data-sets. We found that fHRV complexity in LV/HF vs. HV/LF state in 11 out of 12 fetuses was lower at the time scale of 0.2 s (2.5 Hz) and in 10 out of 12 fetuses higher at the time scale 2.7–3.3 s (Frank et al., 2006a,b) (0.15–0.19 Hz) (Figures 3 and 4).

## Discussion

Here we show that the variation in fHRV complexity due to changes in sympathovagal activity depends on the time scale used for the analysis. This sympathovagal activity is dependent on behavioral states. It is intriguing that these neural influences can be dissected mathematically using an *a posteriori* approach toward estimating fHRV complexity on physiologically relevant time scales.

### Vagal and sympathetic modulations of fHRV impact specific time scales of fHRV

The KLE function allows a more sophisticated detection of state-dependent differences in fHRV complexity. Subtle effects of behavioural states on fHRV time scales retreat and are likely methodically “undersampled” *vis-à-vis* the more pronounced changes induced by the β-receptor-mediated sympathetic blockade and even more so by the vagal blockade. Thus, for the sake of comparing our previous work using aAIF and the current paper we will first focus on the effects of the pharmacological blockades vs. baseline rather than dissecting the baseline into HV/LF or LV/HF ECoG state activities.

Our findings have several implications for understanding near-term fetal sheep physiology of fHRV complexity properties and present possibilities for using fHRV to monitor fetal health.

First, we identified precise time scales of fHRV fluctuations which result from the vagal modulation of fHRV to be 0.2 s (2.5 Hz) and 0.3 s (1.6 Hz). This is in line with previous studies in sheep and human fetuses (Frank et al., 2006a,b; David et al., 2007; Frasch et al., 2007a,b, 2009a,b). These time scales find themselves within the frequency domain equivalent to the upper end of the high frequency spectral power band of fHRV (0.2–2.5 Hz). Of note, atropine blockade of vagal modulation of fHRV resulted in a drop of fHRV complexity at 1.6 Hz and 2.5 Hz compared to the baseline LV/HF ECoG state activity, but not when compared to the HV/LF ECoG state activity. Comparison to the latter rendered a drop in fHRV complexity at 2.5 Hz only, a value high enough to be potentially overlooked by studies designed with certain frequency bands set *a priori*.

Similarly, β-receptor-mediated blockade of the sympathetic modulations of fHRV revealed increases in fHRV complexity at the 0.5–0.6 s time scale (0.8–1 Hz) when compared to LV/HF ECoG state activity, but not when compared to the HV/LF ECoG state activity. While we anticipated the complexity increase following this blockade of sympathetic fHRV modulations based on previous studies (Frasch et al., 2009a,b), the time scale on which we detected this decrease is less intuitive. It may be due to unmasking vagally-mediated fluctuations within fHRV time scales corresponding to a somewhat lower range of high frequency band power spectrum associated with vagal influences on fHRV (Van Leeuwen et al., 2003; Frank et al., 2006a,b; David et al., 2007; Frasch et al., 2007a,b, 2009a,b; Van Leeuwen et al., 2007). This may now explain the physiological mechanism behind our earlier finding that propranolol blockade in these fetal sheep resulted in a selective decrease in the nonlinear part of fHRV complexity on the short-term time scale (i.e., beat-to-beat in that case) (Frasch et al., 2009a,b). The mechanism may be as follows: propranolol-blockade results in a relative increase of vagally mediated fluctuations in fHRV, which we measure here as a complexity increase at ~0.9 Hz. However, measured with aAIF function over all physiologically relevant time scales this sympathetic blockade appears to result in a fHRV complexity decrease—a meaningful finding reflecting an overall reduction in system complexity due to blockade of one of the branches of the autonomic nervous system. Moreover, we showed that vagally mediated fHRV complexity was sufficiently explained by its linear properties (Frasch et al., 2009a,b). In light of this “Gedankenexperiment,” it is conceivable that the pinpointed fHRV complexity increase measured by the KLE function due to sympathetic blockade within the vagally mediated time scale corresponds to a complexity decrease in the non-linear part of fHRV complexity, which represents an emergent component that is due to non-linearly superimposed interactions of vagal and sympathetic fluctuations of fHRV.

Second, fetal sheep and human HV/LF and LV/HF ECoG sleep state dynamics are in contrast regarding vagal and sympathetic contributions to FHR and fHRV (Frank et al., 2006a,b). This should be kept in mind when inferences to autonomic nervous system contributions during respective ECoG states are made in this paper with the intent of comparing them to human fetal physiology. With this in mind, the relatively high frequency time scales of vagal fHRV modulations reported in the present study appear to be in contrast with some human fetal studies near-term (Van Leeuwen et al., 2003, 2007). These studies did not account for fetal behavioral states. Accounting for the behavioral states renders similar ranges of state-dependent fHRV fluctuations (Frank et al., 2006a,b).

Hence, for the first section of our discussion we make two key observations: First, fetal behavioral states should be accounted for when effects of treatments or conditions are studied with fHRV. Second, to account for the behavioral state-dependent fluctuations of vagal and sympathetic modulations of fHRV complexity, *a posteriori* study of fHRV time scales appears to be a more encompassing approach to capture all possible time scale dynamics. This challenge is unique to HRV monitoring during the perinatal stage of development when ~90% of time is spent during HV/LF (NREM) or LV/HF (REM) behavioral states and only ~10% of time is spent in wakefulness (Richardson and Gagnon, 2008).

### Contributions of vagal and sympathetic activities to ECoG state-dependent changes in fHRV complexity

Our results on ECoG state-dependent differences in fHRV complexity provide several insights into what appears to be a species-specific and time scale-specific behavior in sheep and human perinatal brain development.

First, confirming our previous findings in a different set of fetal sheep of the same gestational age, here we also found higher complexity during LV/HF on long-term time scale of 2.7–3.3 s (0.15–0.19 Hz) (Frank et al., 2006a,b). This time scale region corresponds to fHRV low frequency band spectral power (0.04–0.2 Hz) known in sheep fetuses to contain both sympathetic and vagal influences on fHRV (Frasch et al., 2007a,b, 2009a,b). This finding is in physiological contrast to human mature fetuses of comparable gestational age who show an inverse relationship to behavioral sleep states throughout all physiologically relevant time scales (Frank et al., 2006a,b).

Of note, we did not detect a difference in fHRV complexity at the time scale where the complexity increased due to sympathetic blockade (~0.5 s or 0.9 Hz). It is possible that sleep state-dependent fluctuations in sympathetic activity on time scales unaffected by β-receptor sympathetic blockade may contribute to the observed lower complexity in the 0.15–0.19 Hz range. We and others have shown that propranolol blockade results in low frequency spectral power decrease of fHRV (0.04–0.2 Hz) (Yu and Lumbers, 2000; Frasch et al., 2009a,b). However, the precise distribution of spectral power was not described. Hence, it seems advantageous for approaches in frequency and multiscale complexity domains of fHRV analysis to report the most complete possible spectra or time horizons (0–5 s appears to be an adequate choice) instead of limiting the results by reporting *a priori* predefined frequency bands or time scales, respectively.

Second, it is intriguing in this context that in the present study we found—in a seeming paradox—lower complexity during LV/HF ECoG state at the same short-term time scale where complexity is also decreased following vagal blockade (at 0.2 s or 2.5 Hz). At first examination, this should mean that lower fHRV complexity during LV/HF ECoG state is due to a relatively lower contribution (i.e., lack of dominance) of vagal activity to modulations of fHRV complexity at this short-term time scale. A closer look suggests two following interpretations.

On one hand, this finding closely resembles the human fetal physiology of fHRV complexity at a comparable gestational age with regard to this specific time scale. This suggests that the species difference may in fact be maturational and not fundamental (Frank et al., 2006a,b). That is, the time scale-specific species difference may be related to the fine tuning of the state-dependent sympathovagal activation/inhibition patterns and their degree of fHRV modulation. It is known that various organ systems, such as the brain, mature at different speeds in different species (Dobbing and Sands, 1979). In addition, postnatally the autonomic nervous system and cardiovascular controls in lambs appear to behave somewhat similarly to human neonates in relation to sleep states and bivariate heart rate/blood pressure coupling, while the heart rate itself continues to be higher during quiet sleep (~HV/LF ECoG state) versus active sleep (~LV/HF ECoG state) in contrast to human neonates (Silvani et al., 2005; Frasch et al., 2007a,b; Booth et al., 2011a,b). Further comparative research is needed to address the sleep state-dependent differences in bivariate and univariate cardiovascular control patterns found in sheep and human species. Such research will render valuable data to better interpret animal research findings in human physiological and clinical contexts, e.g., in the field of Sudden Infant Death Syndrome (SIDS).

On the other hand, our finding of lower fHRV complexity during the LV/HF ECoG state at the 0.2 s time scale where fHRV complexity is also decreased following vagal blockade is in direct contrast with studies also carried out in fetal sheep near-term that showed a dominance of vagal control of cardiovascular system during LV/HF state with lower FHR and blood pressure values versus HV/LF state (Zhu and Szeto, 1987; Jensen et al., 2009). Of note, these studies did not examine higher order properties of fHRV which contain the information about influences of vagal and sympathetic rhythms on fHRV. In a smaller study (*n* = 6) using the same signal analytical approach and comparable gestational age of fetal sheep as presented here, we could not detect differences between ECoG states at this time scale of fHRV complexity (Frank et al., 2006a,b). We cannot exclude that this may be due to a smaller sample size. Similarly, in this study, we could not detect any differences between the ECoG states regarding fHRV complexity at an equally small sample size of *n* = 6.

We believe this paradox can be resolved in the following complementary ways:

1. In the current study we did not investigate the contribution of non-linearities to the overall complexity as done previously (Frasch et al., 2009a,b). In that study we showed that vagal modulations of fHRV contribute to fHRV linear properties on short-term (beat-to-beat) time scales in near-term sheep, rather than to its non-linear components (Frasch et al., 2009a,b). This means that the vagal blockade-induced fHRV complexity decrease we found here on the same time scale as the fHRV complexity decrease during LV/HF ECoG state vs. HV/LF ECoG state may be due to different contributions of non-linear parts of fHRV complexity to the overall complexity changes we assessed in the present study.
2. Inferring about contributions of the underlying sympathetic and vagal rhythms from pharmacological blockade-induced fHRV complexity changes to observations made during physiological, unperturbed, ECoG state cycling carries an inherent limitation discussed earlier (Frasch et al., 2009a,b). The limitation is the same that led us to deploy complexity analyses of fHRV in the first place, namely, the non-linear nature of the influences sympathovagal fluctuations have on fHRV. The decreases of overall fHRV complexity following vagal and sympathetic blockades that we reported suggest that both vagal and β-receptor-mediated sympathetic modulations contribute to the complexity of fHRV (Frasch et al., 2009a,b). In agreement with this, the concept of reciprocal vagal or sympathetic activation has been challenged by the evidence of nonreciprocal autonomic modulation of HRV (Guzzetti et al., 2005; Paton et al., 2005), suggesting concomitant vagal and sympathetic activation as in a complex network.

In summary, for the second section of our discussion and to address the apparent “paradox,” we suggest that our findings for differences in fHRV complexity during LV/HF vs. HV/LF ECoG states should be seen as reflecting changes in sympathovagal activation patterns as they modulate fHRV, rather than as distinct, independent effects of sympathetic or vagal modulations of fHRV.

### Implications for human FHR monitoring

Our data suggest that relatively short FHR segments of as few as ~600 heart beats per behavioral state appear to be sufficient to allow for state discrimination based on *a posteriori* time scale fHRV complexity analysis. Moreover, R–R intervals with sampling rates obtainable from regular ultrasound- or scalp-electrode derived FHR read-outs should still permit state discrimination at the time scale of ~3 s or 0.17 Hz. This is relevant for the prospect of using antepartum FHR monitoring to detect and grade chronic fetal hypoxia. This might be possible because chronic hypoxia disrupts fetal HV/LF and LV/HF ECoG states and may also have a similar effect on the accompanying fHRV dynamics (Keen et al., 2011a,b). Further studies will be needed to investigate these physiologic and pathophysiologic relations.

### Perspectives and implications

Vagal and sympathetic modulations of fHRV show sleep state-dependent and time scale-dependent complexity patterns.

The pathophysiologically motivated “fight and flight response” paradigm of the “linearizing,” complexity-decreasing effect of sympathetic HRV modulation may be not comprehensive enough to capture the “every day” physiological context. Further studies are needed to enhance this paradigm toward the concept of sympathovagal co-activation, a synergistically occurring physiological pattern of autonomic nervous system activity within the larger multi-organ network. In this context, a unified *a posteriori* multi-scale approach to HRV complexity estimation is required to facilitate cross-study and cross-species comparisons and translation of knowledge into improved HRV monitoring. Ultimately, to more fully account for behavioral state-, time scale- and species-dependent fluctuations in such physiologic patterns, this paradigm should allow for inclusion of multivariate data-sets such as ECoG and blood pressure signals.

### Conflict of interest statement

The authors declare that the research was conducted in the absence of any commercial or financial relationships that could be construed as a potential conflict of interest.
